# Radiation Treatment for WHO Grade II and III Meningiomas

**DOI:** 10.3389/fonc.2013.00227

**Published:** 2013-09-02

**Authors:** Brian P. Walcott, Brian V. Nahed, Priscilla K. Brastianos, Jay S. Loeffler

**Affiliations:** ^1^Department of Neurological Surgery, Massachusetts General Hospital, Harvard Medical School, Boston, MA, USA; ^2^Department of Medicine, Massachusetts General Hospital, Harvard Medical School, Boston, MA, USA; ^3^Department of Medical Oncology, Dana-Farber Cancer Institute, Boston, MA, USA; ^4^Broad Institute of Massachusetts Institute of Technology, Harvard University, Boston, MA, USA; ^5^Department of Radiation Oncology, Massachusetts General Hospital, Harvard Medical School, Boston, MA, USA

**Keywords:** meningioma, radiosurgery, proton therapy, surgery, brachytherapy

## Abstract

The treatment of meningiomas is tailored to their histological grade. While World Health Organization (WHO) grade I lesions can be treated with either surgery or external beam radiation, WHO Grade II and III lesions often require a combination of the two modalities. For these high-grade lesions, conventional external beam radiation is delivered to either the residual tumor or the surgical resection margin. The optimal timing of radiation, either immediately following surgical resection or at the time of recurrence, is yet to be determined. Additionally, another method of radiation delivery, brachytherapy, can be administered locally at the time of surgery for recurrent lesions. Altogether, the complex nature of WHO grade II and III meningiomas requires careful treatment planning and delivery by a multidisciplinary team.

Meningiomas are classified morphologically by the World Health Organization (WHO) grading scheme into three categories (1). While most meningiomas are benign or grade I, the propensity of certain subtypes to recur following treatment creates a pathological course that can lead to a spectrum of malignant disease.

The goal of surgery is to safely resect the tumor and obtain a diagnosis and pathological grade. Subsequent radiation therapy depends on the extent of resection and pathological characteristics. Patients harboring either WHO grade II or III meningiomas have higher recurrence rates, varying between 29–52 and 50–94% respectively (2–9). This may be due in part to parenchymal invasion or the aggressiveness of residual tumor cells. Higher recurrence rates correlate with decreased overall survival (6). The role of surgery alone is typically inadequate given the high local recurrence rate. Preliminary observational studies have demonstrated that radiation improves recurrence rates and overall survival in these cases (10–12). Therefore, for grade II and grade III lesions, radiation is often an integral part of any treatment regimen. Radiation is delivered via an external beam source or via radioactive seeds implanted locally during a surgical procedure (brachytherapy).

## External Beam Radiation

Several types of external beam radiation exist, including newer technologies and delivery techniques such as photon based stereotactic radiosurgery (13–16), hypo-fractionated radiation therapy (17, 18), and hadron therapy (e.g., proton radiation) (19–22). Treatment plans may be devised to target the remaining lesional tissue following subtotal resection, or the resection cavity plus a margin of approximately 1 cm following gross total resection of higher grade tumors. Radiation treatment planning requires balancing the dose and volume delivered for a clinical benefit with the potential toxicities. Radiation necrosis (22), exacerbation of peritumoral edema (23–25), optic neuropathy (26), cranial nerve palsy (27), and wound complications are the principle complications seen following radiation therapy of intracranial meningiomas.

While the literature is rich with studies on meningiomas, there is little outcome data available on radiation treatment for WHO grade II and III meningiomas. All current evidence stems from retrospective case series, which have been subject to evolution in treatment methodologies and changes in the grading scheme over time (1, 28). Nonetheless, radiation therapy, typically administered as an immediate adjuvant dose (alternatively at the time of recurrence) is common for the treatment of these lesions. It should be noted that since there are no randomized trials, controversy and differential practice patterns exist with several aspects of radiation therapy including timing following resection, and best modality of radiation therapy.

Based upon retrospective data, there is a trend toward improved outcomes with immediate post-operative radiation following gross total resection with WHO grade II and III meningiomas. A single center series analyzed outcomes in 108 patients who underwent a gross total resection (Simpson grade I) for an atypical meningioma (2). In this series, the vast majority of patients (100 patients) were treated with surgery alone, while only 8 patients received immediate adjuvant radiation therapy (stereotactic fractionated radiotherapy) to an approximately 1 cm margin around the resection cavity. Overall, 30 patients recurred after gross total resection without adjuvant radiation therapy, whereas none of the 8 patients who received adjuvant radiation therapy recurred. Despite this, rates of recurrence between post-operative irradiated and non-irradiated patients did not reach statistical significance. For the entire cohort, the actuarial recurrence rates at 1, 5, and 10 years were 7, 41, and 48%, underscoring the propensity of these lesions to recur. Disease-specific survival after first recurrence was 86 and 69% at 5 and 10 years, respectively.

In comparison to grade II meningiomas, grade III meningiomas have a more dismal prognosis, as illustrated in several case series. In a group of 13 patients with WHO grade III meningiomas who underwent surgical resection, recurrence occurred in 92% of patients at a time interval of 0.4–2.8 years (29). The 5- and 8-year actuarial survival in this group was 47 and 12%, respectively. Only three of the initial cohort received adjuvant radiation therapy following primary resection. In another study of grade III meningiomas, the 5- and 10-year survival rates were found to be slightly higher at 64.3 and 34.5%, respectively (9). Despite the aggressive nature of these tumors, adjuvant radiation therapy is not routinely administered. One survey reported that only 9 of the 56 studied centers recommended radiation after gross total resection of an atypical meningioma (30).

Undoubtedly, treatment plans for patients are individualized and based on a multitude of factors. Recent elucidation of the genomic landscape of these lesions has identified several genetic subtypes of tumors that may prove to have distinct clinical characteristics and even the potential for response to targeted therapeutics (31–33). Additionally, atypical meningiomas (WHO grade II) with osseous involvement are associated with poorer outcomes. In 47 patients with atypical meningiomas treated at our institution, bony involvement was associated with an increased rate of disease progression and decreased survival (34). Therefore, bone assessment radiographically and histologically is important, and further studies should assess the effectiveness of bone resection and/or targeted radiation therapy to the bone to improve outcome.

## Brachytherapy

Brachytherapy, the local implantation of a radiation source, is considered “salvage” therapy for the recurrence of aggressive atypical and anaplastic meningiomas. At the time of re-operation, radioactive sources or “seeds” of iodine-125 are implanted in the resection cavity in an array that generally generates a median total activity of between 20 and 60 mCi. Success has been reported with this type of radiation treatment modality, with early reports of two patients with recurrent malignant meningiomas having long term remission after interstitial brachytherapy (35). The largest series to date (21 patients) reported a median survival following implantation of 1.6 years for atypical meningioma and 2.4 years for anaplastic meningioma (36). In this same series, a very high complication rate was reported, with 27% of patients experiencing radiation necrosis and 27% with wound complications requiring re-operation.

## Ongoing Studies

Several trials are studying the role of radiation therapy in the management of patients with atypical or anaplastic meningiomas. Some studies are evaluating differences in radiation delivery modalities and techniques, such as UPCC 24309 (ClinicalTrials.gov Identifier: NCT01117844). In this trial, outcomes from proton beam therapy will be compared to historical controls associated with conventional photon beam treatment. Other studies aim to determine the efficacy of immediate post-operative radiation therapy of meningiomas following resection. One trial conducted by the Radiation Therapy Oncology Group (protocol RTOG 0539, ClinicalTrials.gov Identifier: NCT00895622) is monitoring low-grade meningiomas (WHO Grade I) with clinical observation following initial surgery, while those with intermediate or high-risk disease (such as all WHO grade II and III meningiomas) receive 6 weeks of radiation therapy using either three-dimensional conformal RT or intensity-modulated radiation therapy.

**Figure 1:**
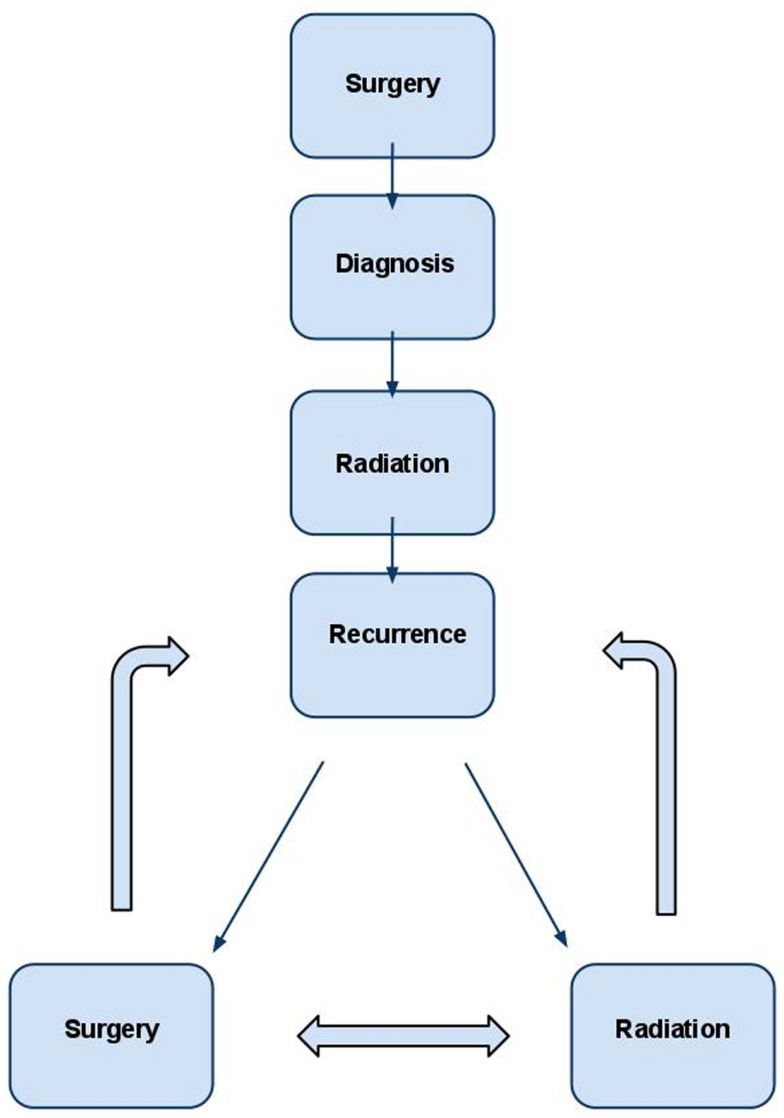
**Management strategy for WHO grade II and III meningioma**. In the typical patient, diagnosis is first obtained by surgery. Post-operative radiation is administered to decrease the likelihood of recurrence in many cases and almost universally in cases of subtotal resection. If recurrence occurs, retreatment with radiation and/or surgery are both viable options and should be individualized based on the unique clinical scenario.

The other study, run by the European Organization for Research and Treatment of Cancer (protocol EORTC 22042, ClinicalTrials.gov Identifier NCT00626730), has patients with atypical or malignant meningioma being treated with adjuvant radiation therapy following surgical resection. While we eagerly await the results of these trials to optimize patient care, current management of patients is based on the best evidence available. While randomized trials do not exist, adjuvant radiation therapy immediately following initial surgery for WHO Grade II and III meningioma should be considered given the high rate of local recurrence (Figure 1). Radiation should also be administered for patients undergoing subtotal resection. These recommendations are congruent with National Comprehensive Cancer Network Clinical Practice Guidelines in Oncology (37).

### Case illustration 1

**Figure 2:**
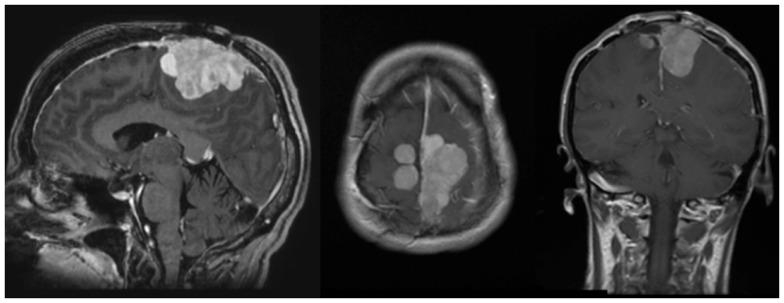
**Pre-operative MRI**. MRI (l–r, sagittal, axial, coronal) demonstrates an avidly enhancing, complex falcine meningioma involving both sides of the superior sagittal sinus.

**Figure 3:**
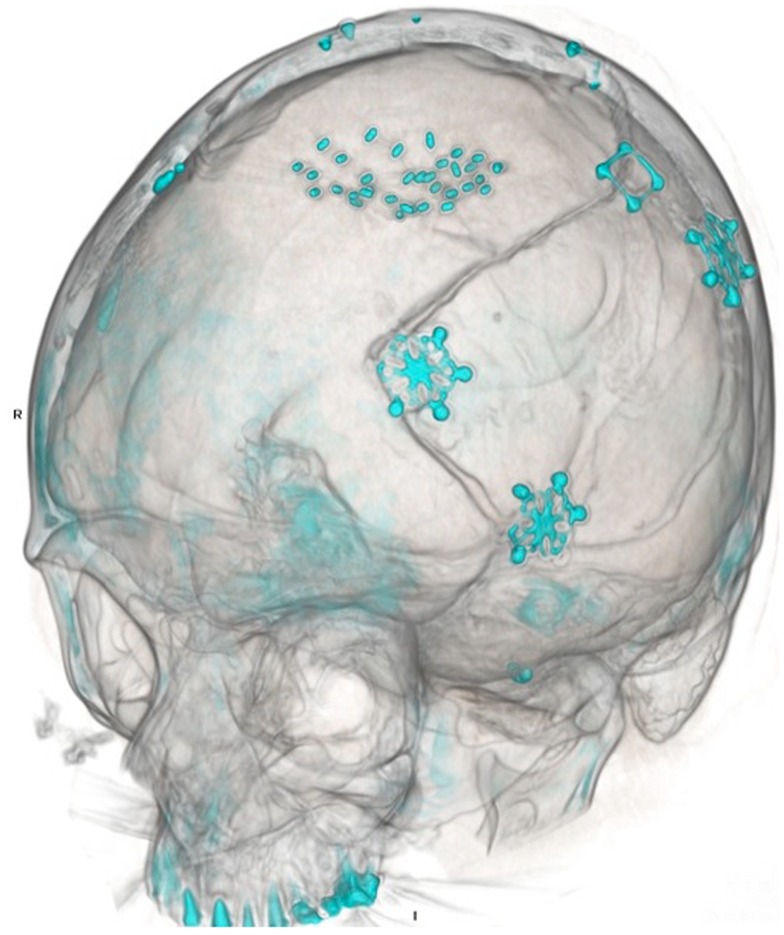
**Post-operative CT scan**. Three-dimensional reconstruction demonstrates both prior craniotomies with microplate fixation, as well as radioactive brachytherapy seeds placed in the resection cavity.

**Figure 4:**
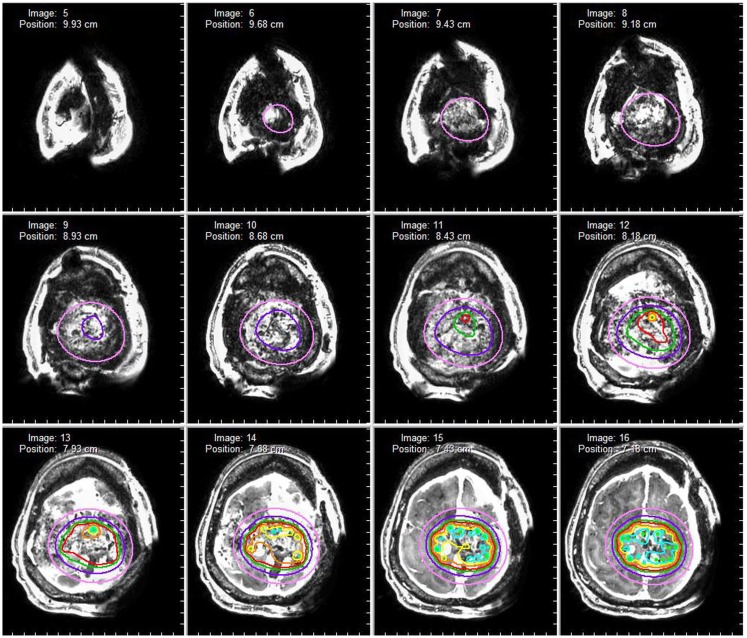
**Dosimetry map**. A dosimetry map of the radioactive brachytherapy seeds is constructed based on seed arrangement in three-dimensional space. The red isodose line represents a prescription dose 80 Gy (80% isodose level) whereas the pink isodose line represents a prescription dose of 25 Gy (25% isodose).

A 50-year-old female initially presented 8 years prior with a 5 cm × 5.5 cm × 5 cm enhancing parasagittal lesion with osseous invasion and left sided motor weakness. She underwent a subtotal resection; the pathological diagnosis was consistent with meningioma WHO grade I with no atypical features and a MIB index of 5% (38). Postoperatively, her weakness resolved, but 2 months later she was found to have a new nodular enhancing component in the inferior aspect of the resection site measuring 1.5 cm × 1.5 cm × 1.3 cm. She underwent intensity-modulated radiation therapy 6000 cGy without complication. Six years following radiation, she presented with right-sided weakness and was found to have very aggressive interval growth of the residual tumor (Figure 2). Given the suspicion that her tumor had undergone either atypical or anaplastic transformation, she underwent subtotal resection and placement of interstitial brachytherapy I(125) seeds (Figures 3 and 4). Pathological diagnosis of the tumor removed at the time of the second surgery revealed an atypical meningioma, WHO Grade II of III with high cell density, architectural sheeting, and prominent nucleoli. This case highlights the unique scenario of tumor of progression following treatment. When atypical or malignant progression is suspected on pre-operative imaging, brachytherapy can be considered at the time of re-operation.

### Case illustration 2

**Figure 5:**
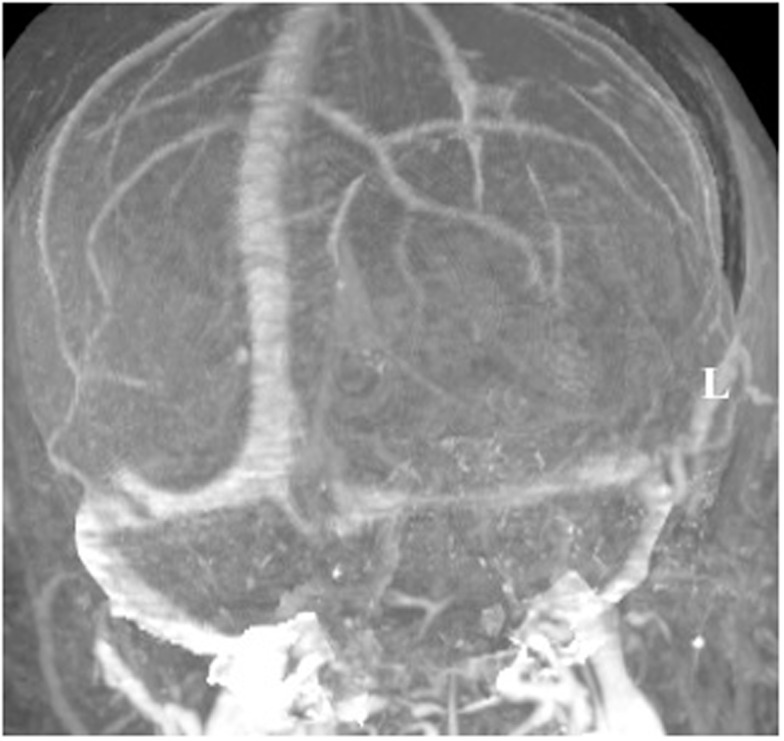
**MRI venogram**. An MRI venogram demonstrated that the left transverse sinus was atretic and the right transverse sinus was dominant.

A 60-year-old woman underwent gross total resection of her right frontal convexity meningioma; and received post-operative radiation for the pathological diagnosis of atypical meningioma. Two years later on routine follow-up, she was found to have a new lesion invading the torcula. An MRI venogram was obtained that demonstrated an unfavorable dural sinus configuration; her left transverse sinus was atretic and her right transverse sinus was dominant (Figure 5).

**Figure 6:**
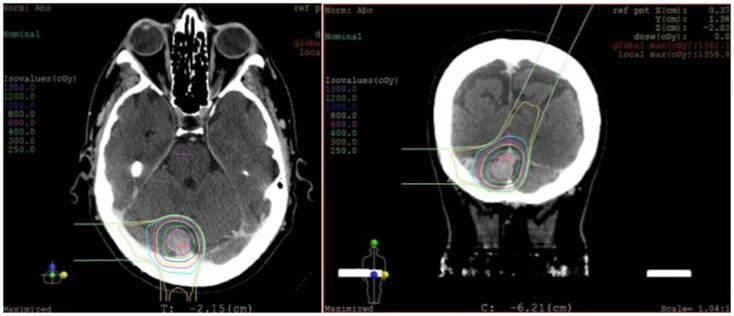
**Radiosurgery dosimetry**. Dosimetry map for proton radiosurgical treatment of a torcular meningioma.

In weighing the risks and benefits of open surgery versus stereotactic radiosurgery, it was decided that radiosurgery would be the best treatment option for her. She underwent stereotactic radiosurgery to the lesion, presumed to be an atypical meningioma based on her previously pathology from the first surgery (Figure 6). From a technical aspect, given the sinus involvement on pre-operative imaging, it was likely that a gross total resection would not be able to be achieved and any tumor left remaining on the sinus would need to be treated with radiation, regardless. Stereotactic radiosurgery provides the benefit of treating the entire lesion while minimizing the risk of a potentially catastrophic venous infarct or hemorrhage associated with open surgery.

## Conclusion

World Health Organization grade II and III meningiomas can have a malignant clinical course. Multidisciplinary strategies of care involving aggressive surgical resection and post-operative radiation therapy may reduce recurrence rates. Studies to assess the optimal timing and modality of post-operative radiation are underway.

## Conflict of Interest Statement

The authors declare that the research was conducted in the absence of any commercial or financial relationships that could be construed as a potential conflict of interest.
